# Community-Institutional Partnerships to Strengthen Maternal Health Care: Case Study of the First Obstetrics and Gynecology Specialty Training Program in Liberia

**DOI:** 10.3389/fpubh.2021.779035

**Published:** 2022-02-07

**Authors:** Ann Marie Beddoe, Maureen Reis, Angela Benson, Lise Rehwaldt, John Mullbah, Janetta Johnson, Molly Lieber, Andrew Dottino, Corrine Maund, Sara Campbell, Vanessa Kerry, Julie Solomon, Whitney Lieb, Michael Brodman, Etedafe Gharoro, Sadath Sayeed, Tej Nuthulaganti, Billy C. Johnson, Jerry Brown, Roseda Marshall, Bernice Dahn

**Affiliations:** ^1^Icahn School of Medicine, New York, NY, United States; ^2^Global Health Service Partnership, Washington, DC, United States; ^3^Seed Global Health, Boston, MA, United States; ^4^Liberian College of Physicians and Surgeons, Monrovia, Liberia; ^5^Harvard Medical School, Boston, MA, United States; ^6^Massachusetts General Hospital, Boston, MA, United States; ^7^Weill Cornell Medicine/New York-Presbyterian, New York, NY, United States; ^8^John F. Kennedy Medical Center, Monrovia, Liberia; ^9^Clinton Health Access Initiative, Cambridge, MA, United States; ^10^University of Liberia, Monrovia, Liberia

**Keywords:** health system strengthening, post-graduate education, maternal health, faculty mentoring, community-institutional partnership

## Abstract

Despite major setbacks to its health infrastructure and health workforce capacity, Liberia began its first post-graduate training program for physicians in 2013. Specialty training in Internal Medicine, Pediatrics, General Surgery and Obstetrics and Gynecology were the four inaugural Residency programs that recruited graduates from the country's only medical school, A.M. Dogliotti College of Medicine. The Obstetrics and Gynecology residency program was designed to combat the rising maternal mortality and strengthen health systems to improve maternal care. The program adapted in the face of challenges posed by limited financial support, lack of specialist-faculty and general physician shortages and the Ebola virus outbreak. The manuscript discusses the challenges and successes of the program and demonstrates how the shortage of teaching faculty was addressed by developing a collaboration between local government and educational communities, a United States (US) academic institution and volunteers from the Global Health Service Partnership.

## Introduction

Liberia suffered through 14 years of conflict and two civil wars from 1989 to 2003. During that time all health-related infrastructure including the only medical school was severely damaged. Loss of vital school facilities, and an acute shortage of teaching faculty who fled the country for fear of losing their lives, resulted in an almost complete cessation of the medical school that graduated only 17 students between 1999 and 2002 (1). A post war survey among medical students revealed that all students experienced delays; 86% of them cited the war as the main cause of delay in their medical education, while 75% cited lack of faculty as a major contributor (2). The war took an immense toll not only on health infrastructure but on the much needed health workforce capacity of the country.

Although every aspect of health care in Liberia was affected, maternal and neonatal care were among the hardest hit. With only 51 doctors left in the country after the war, and rising maternal and neonatal mortality rates, a program initially piloted during the war continued to provide post-internship training in the emergency obstetric care (EmOC) (3). The Ministry of Health and Social Welfare through a human resources plan extended and expanded this training at the end of the war to train nurses and physician assistants to manage emergency obstetric and neonatal care (EmONC) (4). Despite success with these programs, gaps remained. Maternal mortality of over 994/100,000 live births and a paucity of reproductive health specialists were some of the factors that motivated the administration to invest in specialty training of physicians in maternal health (5, 6).

## Context

By 2012 of the estimated 117 physicians registered by the Liberian Medical and Dental Council, only 51 were Liberian trained physicians; the majority were expatriates returning to help rebuild Liberia's health and medical education sectors. Most of the Liberian-trained physicians were employed in general practice, public health, or administrative positions; fewer than 15 were specialists (7, 8). In 2013, the ratio of physicians to population was approximately 0.3 per 1,000 population (9), which was lower than the mean global physician density of 1.3 per 1,000 population needed to provide effective service coverage and below 1 physician per 1,000 population currently recommended by the WHO (10, 11). With patients having to travel abroad for specialty care, maternal mortality increasing, and a lifetime risk of maternal death of 1 in 24 (12), the Ministry of Health launched the country's first post graduate training programs including residency training in Obstetrics and Gynecology.

## Key Programmatic Element

Under the leadership of then Deputy Minister of Health, Bernice Dahn, a Technical Working Group (TWG) of senior Liberian physicians convened in 2011 to set the parameters and establish realistic goals for developing the first specialty training programs in Liberia. Early collaborators to this program were the West African College of Physicians and Surgeons, the Ghana College of Physicians and Surgeons and the Ministry of Health of the Republic of Liberia.

### Exploratory Phase

The TWG visited Ghana to tour the Ghana College of Physicians and Surgeons. The first collaborative meeting with representatives of the Ghana College was held in Ghana in mid 2012.

### Action Phase

Upon returning to Liberia and with the guidance from Ghana, the West African College of Physicians (WACP) and the West African College of Surgeons (WACS), the TWG drafted the First Act of the Liberian Residency training program (8). Specialty training programs in Internal Medicine, Surgery, Pediatrics and Obstetrics and Gynecology were selected to address the gaps in the most essential services that were unavailable in the country (13, 14). The group mobilized community support and advocacy, completed their strategic planning and introduced the Liberian Residency Training Act to Parliament which was unanimously passed. The Liberian College of Physicians and Surgeons, an autonomous agency of the Government of Liberia, was thus created in 2012, as the accreditation body to oversee post-graduate training and work closely with the A.M.Dogliotti College of Medicine to provide a pipeline for recruiting residency candidates. The World Bank provided the first year of funding for the program; prolonged negotiations for this funding resulted in a delayed start to the Residency programs from July to September 2013.

### External Academic/Institutional Partnerships

Mount Sinai Hospital has had a longstanding relationship in Liberia in the field of women's health. In 2007 at the annual Clinton Global Initiative meeting in New York, key-note speaker President Ellen Sirleaf appealed for help to rebuild Liberia's health sector citing the country's lack of physicians. In 2008, twenty-eight health workers from the Mount Sinai System, among them three senior Ob/Gyn faculty were deployed to Monrovia and Bong County. The team introduced daily bedside rounds and assisted in outpatient and in-patientcare of patients. This began a long- term relationship in the field of maternal health at John F. Kennedy (JFK) Hospital in Monrovia and Phebe Hospital in Bong County. As plans to establish the post graduate program evolved, the Ministry of Health called upon Mount Sinai to assist with the development of the obstetrics and gynecology residency program. The Global Health Service Partnership (GHSP), a collaboration between the Peace Corp and SEED Global Health was established to provide health care and education in areas of the world with extreme shortages of healthcare providers (15). Given the fixed finances for implementation of the program and anticipating the need to increase the number of faculty as the residency training expanded, the Minister of Health reached out to the Peace Corp and GHSP to provide additional teaching faculty for the obstetrics and gynecology residency.

#### Purpose

This manuscript explores the challenges and successes of this multi-institutional collaboration in the first 4-years of the residency training program in Obstetrics and Gynecology in Liberia, following the program from its inception in 2013 to the graduation of the first cohort in 2017.

## Methods

### Program Concept

The residency program, originally proposed as a 4-year training program was changed to a 3-year program following the Ebola outbreak. A 9-month delay caused by the outbreak resulted in the first cohort completing their first year of training (PGY-1) over a 21-month period (September 2013-June 2015). The outbreak, and the urgency for maternal health specialists were some of the factors that influenced that change.

JFK Hospital the largest tertiary hospital in the city of Monrovia was designated as the primary training site for the program. Redemption Hospital, the largest public hospital in the city of Monrovia was selected as the rotation site in the city, where residents covered day services only. Rotations at Phebe Hospital in Bong County and Jackson Doe Hospital in Nimba County were identified as the rural rotation sites.

The process of accreditation for designating JFK Hospital as the primary training institution was conducted by an external team of evaluators from the West African College of Surgeons in conjunction with the Liberian College of Physicians and Surgeons at a site visit. Because of the urgent need for specialists in the country the hospital was given partial accreditation. The site reviewers granted authorization to start the training programs with specific recommendations made for upgrading the hospital to obtain full accreditation.

### Candidate Recruitment

Residency candidates were recruited from among graduates of the AM Dogliotti College of Medicine and non-specialized physicians practicing throughout Liberia. A selection examination was administered, and candidates were called for interviews based on scores. Five candidates were selected based on examination scores and interview performance. The maximum number of recruits was capped at 5 per year, to ensure that the pool of graduating physicians available to provide service throughout the country was not diluted. To avoid the risk of “brain drain” the first cohort of recruits included previously graduated physicians with long standing roots in Liberia, while subsequent cohorts gave more consideration to recent graduates from the AM Dogliotti College of Medicine (16). All candidates selected into the program either continued their employment with the Ministry of Health or became employees of JFK Hospital.

### Faculty Recruitment

Recruitment of faculty was coordinated by the Liberian College of Physicians and Surgeons (LCPS). Liberian physicians who completed residency training in obstetrics and gynecology abroad, and Board-Certified physicians from Mount Sinai Medical Center were interviewed and offered employment through the LCPS. Volunteer candidates from the Peace Corp were interviewed and selected through an application process led jointly by the LCPS, the Global Health Service Partnership (GHSP) and Mount Sinai School of Medicine. The ratio of faculty to trainees was set at 1:3 following the guidelines of the West African Colleges of Physicians and Surgeons.

### Curriculum Design

The curriculum was based on a modification of the Ghanian curriculum, adapted to meet the needs of Liberia, and harmonized with the West African College curriculum (17). Because the majority of the incoming first year residents were trained before the war, it was necessary to include a remedial basic science review for the first 6-months of the program. The goal was to gradually phase out the basic science review and incorporate basic sciences into the weekly didactics, as more recent graduates from the medical school were recruited. The curriculum was proposed to have rotations for the inaugural first years scheduled in 6-month blocks, rotating between obstetrics and gynecology at JFK and Redemption Hospitals. In the second year of the program PGY-2 residents were to be assigned to Phebe Hospital for the entire year, while incoming first years followed the above PGY-1 schedule. Third year residents were to spend the year providing senior level service at JFK and Redemption Hospital (mentoring the incoming first year residents), fulfilling elective requirements that included general surgery, neonatal ICU, radiology, urology, family planning and completing their rural services in Nimba County (Table 1). All residents were required to keep a log-book detailing their surgical cases and complex cases that were presented for discussion.

**Table 1:** Proposed curriculum.

**Proposed curriculum**					
Basic Science Review Topics Covered (First 6 months)					
Month 1	Month 2	Month 3	Month 4	Month 5	Month 6
Embryology Anatomy	Physiology Endocrinology	Biochemistry Cell Biology/ Genetics	Hematology Pathology	Pharmacology Microbiology	Immunology Genetics
Year 1					
Location: Monrovia—Redemption Hospital & JFK Medical Center					
Months 1–6		Obstetrics (prenatal, intrapartum, postpartum) Emergency Room Labor & Delivery Optional: PM remedial Basic Science		Clinical: AM Morning Report Case Discussion Hospital Night call at JFK Medical Center only	
Months 6–12		Gynecology—out-patient, in patient, surgery Surgical Obstetrics		Academic: Journal club & resident presentations	
Year 2					
Location: Bong County—Phebe Hospital					
Months 1–6		High risk Obstetrics Surgical Obstetrics & Gynecology, Emergency Room (coverage for all services)		Clinical: AM Morning Report Case Discussion	
Months 6–12		High risk Obstetrics Surgical obstetrics, Family Planning Gynecology			
Year 3					
	Location: Monrovia—JFK Hospital				Location: Harper - JJ Dossen Hospital
Months 1–12		Chief Resident 2-month rotations Neonatal ICU General Surgery Urology			Junior Residents: Community Obstetrics & Gynecology

### Performance Evaluation

Written examinations were given at mid-year and end of year. Residents who scored below 70% had an opportunity to meet with faculty to review overall topics that were problematic on the examination, and they were then allowed to repeat the examination. An assessment form was completed by faculty and the evaluation was based on residents' performance in patient care, medical knowledge, case presentations, clinical decision making, clinical skills, and surgical skills. In-person evaluations were conducted by faculty with each resident and included a review of the resident's case list and discussion based on their assessment forms. This was followed by a joint meeting with faculty and residents where residents could provide feed-back or discuss any issues that came up during their training that needed attention. To graduate, residents had to sit and pass both a written and an oral examination. Residents who failed the written examination were not allowed to sit the oral exams, until the next orals examinations were scheduled the following year, thus delaying their graduation. Oral examinations were conducted by an examiner from the West African College as the lead examiner. The external examiner reviewed each residents written examination prior to their orals, and then led the exam in collaboration with local faculty. The oral examinations were presented as an objective structured clinical examination (OSCE) that included simulations, interpretation of sonograms general knowledge and case management.

## Results

Between 2013 and 2017 three cohorts matriculated into the residency training program (Table 2). Candidates were recruited from 6 of the 15 counties in Liberia, but most of the candidates were from Monrovia (Montserrado County). There were 13 males and 2 females and the average age at entry into the program fell from 47.8 years (1st cohort) to 34.4 years (3rd cohort).

**Table 2:** Residency cohort demographics.

**Year**	**Age Range (years)**	**Gender Breakdown**	**County of Residence**	**Number of years post- Graduation**	**Prior Employment**
2013–2015	41–56	3 Male 2 Female	3 Mont- serrado 1 Lofa 1 Nimba	4–18 years	4 MOH 1 Private
2015–2016	35–55	4 Male 1 Female	2 Mont- serrado 1Grand Bassa 1 Nimba 1 Sinoe	1–18 years	3 GOL/MOH 2 GOL/JFK
2016–2017	27–43	5 Male	3 Mont-serrado 1 Margibi 1 Nimba	2 years	2 GOL/MOH 3 GOL/MOH

*MOH, Ministry of Health; GOL, Government of Liberia; JFK—John F. Kennedy Hospital*.

At the start of the program there were 2.5 full time equivalents (FTEs) for 5 in coming residents (PGY-1). At the completion of the PGY1 year (21 months, due to delay caused by Ebola) there were 4 residents, one succumbing to Ebola. Post-Ebola PGY1 residents were moved to Bong County where the Ebola outbreak had declined. At the start of their second year of training, these residents remained in Bong County until completion of their PGY2 year. By the time the first cohort graduated in 2017 there were 3.5 FTEs and a total of 14 residents (Table 3).

**Table 3:** Post-graduate rotations/post residency employment.

**Year**	**# Residents at start**	**# Residents at end year**	**# Faculty**	**Faculty: resident ratio**	**Deployment/post residency employment**
Cohort 1
9/2013-6/2015 PGY-1	5	4	2.5 FTE	1:2	JFK Medical Center Redemption Hospital
7/2015- 6/2016 PGY-2	4	4	2.5 FTE		Phebe Hospital
7/2016- 6/2017 PGY-3	4	4	3.5 FTE		Electives+ Community Service
Post Residency Employment	4	n/a	n/a	n/a	3—Ministry of Health 1—Catholic Hospital
Cohort 2
7/2015-6/2016 PGY-1	5	5	2.5 FTE		JFK Medical Center Redemption Hospital
7/2016-6/2017 PGY-2	5	5			Phebe Hospital
Cohort 3
7/2016-6/2017 PGY-1	5	5	3.5FTE	1.4	JFK Medical Center Redemption Hospital

*PGY, Post graduate year; FTE, full time equivalents*.

Performance assessment was based on a modification of the ACGME Obstetrics and Gynecology Milestones. All four graduating residents achieved comprehensive understanding of normal and abnormal, complex antepartum problems including management and recognition of diabetes, hypertension and pre-eclampsia. Residents self-reported confidence in managing obstetrical emergencies and intrapartum problems that covered a range of atypical conditions including, eclampsia, sepsis, malaria, HIV and non-communicable diseases in the patients with and without prior ante partum care, as well as performing inductions and delivery of breech and multiple gestations. They were able to supervise junior residents through primary, secondary Cesarean sections, communicating with midwives, families and hospital staff in a professional manner. Third year residents were adept at routine Gynecologic open procedures, while first, and second year residents achieved the milestones set by the program and felt they had supervision and guidance from their senior residents and faculty.

Resident feedback highlighted lack of subspecialty exposure and clinical/surgical management of oncology, urogynecology and infertility patients, training in endoscopic procedures, vaginal surgery. They cited lack of multidisciplinary support for acutely ill patients requiring intensive care and shortage of supplies/equipment that limited their ability to practice evidence based medicine. Logistical feedback related to the lack of telecommunication services within the hospital necessitating use of their personal phones to address all emergencies. The lack of a cafeteria on campus necessitated residents going off campus for meals and affected their response time if called back for emergencies. All residents had strong positive feed-back regarding the learning experience gained at morning report, scheduled didactics and contact with faculty as well as the community experiences they gained at the rural hospitals.

By the end of the 2016–2017 academic year, all four residents who matriculated in 2013 passed their written and oral examinations on their first attempt allowing them membership into the Liberian College of Physicians and Surgeons. Two residents passed their WACS primary examinations, and all five first year residents passed their final examinations allowing them to become second year residents.

**Figure 1:**
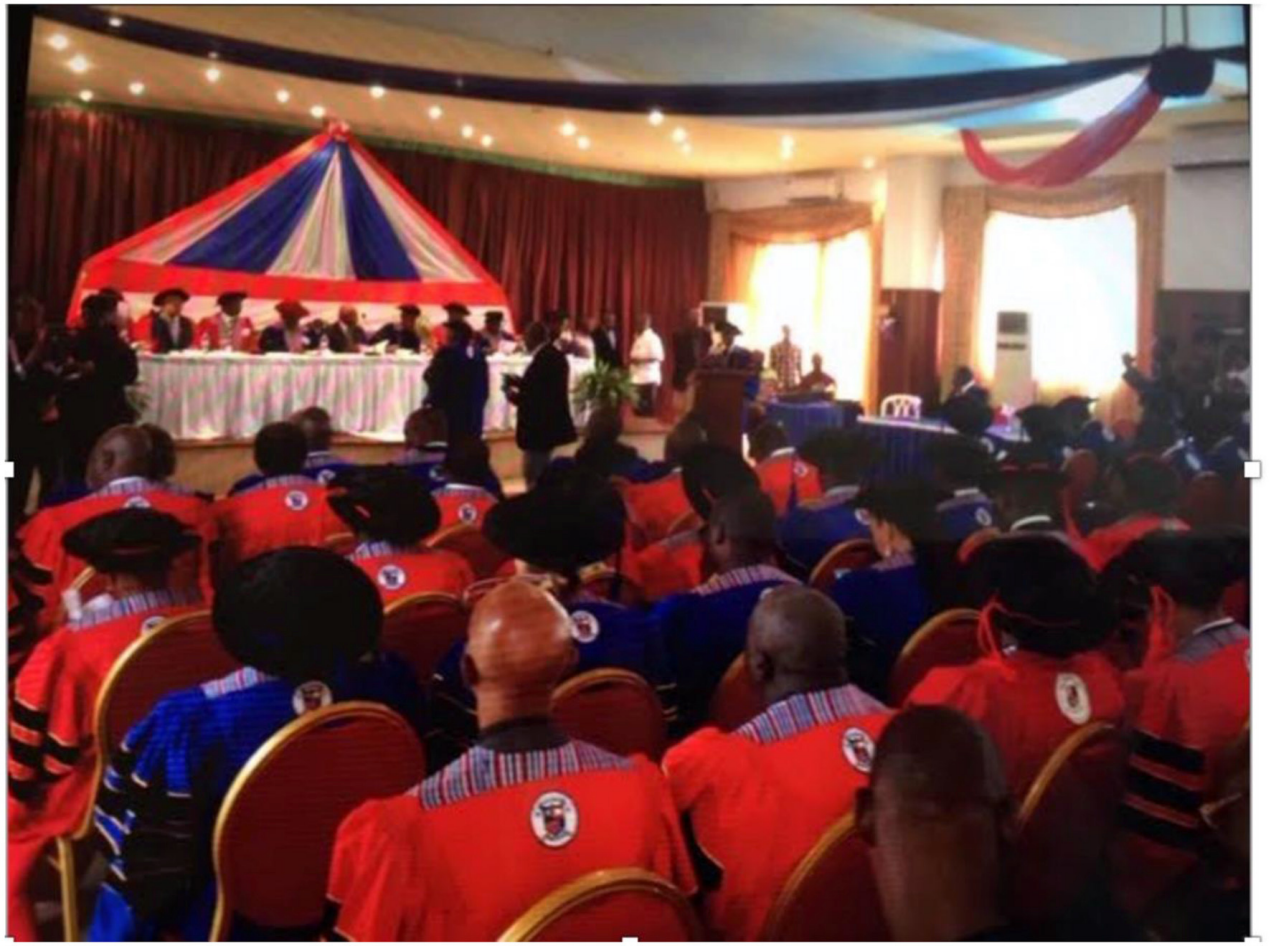
Historic residency graduation September 15, 2017. Graduates in red (shown in front) Ob/Gyn graduating residents, Faculty in Blue: Fellows of the West African College of Physicians, Faculty in Red: Fellows of the West African College of Surgeons.

September 15, 2017, marked the graduation for residents from all four residency programs started in 2013. The residents who competed their training in Obstetrics and Gynecology were the first Liberian trained specialists to have graduated from an in-country program (Figure 1).

## Discussion

Influenced in part by globalization, transnational health networks and advances in science and technology, medical specialization has increased in low- and middle-income countries over the last decade (18). Specialists play an important leadership role in the strengthening of health systems and in advancing evidence-based patient management. In Liberia, a new era of medical education began in 2013 when the first post-graduate specialty training program was inaugurated under the auspices of the local Liberian community that included the Ministry of Health, and local physicians, most of whom became senior members of the newly formed Liberian College of Physicians and Surgeons. Until that time most students graduating from the only medical school in Liberia, A.M. Dogliotti College of Medicine, spent one year of rotating internship at the public hospitals in Liberia, before being assigned to posts across the country to provide care, sometimes as the only physician in a district. One of the goals of the residency program was to allow the rural doctors to communicate with the newly trained specialists to provide consultations on complex cases and improve overall maternal health.

The curriculum and design of the residency programs in Liberia were guided by the tenants of the West African College of Physicians and the West African College of Surgeons. These sister organizations are the accrediting bodies of post graduate training in West Africa promoting education, research, and training in both surgical and non-surgical specialties (19, 20). The paucity of teaching faculty as the number of residents in the program increased was averted by establishing a partnership with the GHSP. Global Health Service Partnership volunteers were recently graduated specialists who functioned as junior attendings and played an important role in bridging the gap between residents in training and senior faculty. They became fully engaged in all aspects of patient care and resident education and were an integral part of the teaching staff.

The first Global Health Service-volunteer completed 1 year and 6 months in Liberia working primarily at Phebe Hospital in Bong County and was succeeded by a second volunteer in 2018. The Global Health service volunteers additionally played an important role in preparing the graduating residents for their exit exams and participated in the ceremony honoring the first Liberian physicians to be trained as specialists in the country. The collaboration between the Liberian medical community, Mount Sinai and the Global Health Service Partnership resulted in an over 50% increase in specialists in the field of Obstetrics and Gynecology in Liberia beginning in 2017.

Despite the success of the program, there were additional challenges facing the residency program that ranged from lack of infrastructure, shortage of supplies, and limited faculty. Basic infrastructural hurdles alluded to in the residents' feedback, were the lack of land-line communication throughout the hospital, lack of on-call rooms and cafeteria facilities for staff. This posed several problems including difficulty in reaching residents who left campus for meals or showers. Because candidates initially entering the program had not previously worked in the structured environment of a residency training program, it was initially challenging to introduce the concept of team-work and shared responsibility and accountability. However, as the program has now trained seven cohorts of residents, there has been a seamless transition that engages responsibility and accountability for the improvement of patient care.

Because there was only one obstetrician at JFK Hospital prior to the residency program, most deliveries were performed by nurse midwives and the introduction of residents into the system initially created some tension. Historically, all first antepartum visits were seen and followed by nurse midwives, with physician involvement reserved for complications that occurred during labor or post-partum. Involving the midwives in conferences where patient management was discussed and having residents working as allies for the best patient outcomes, have gradually changed the dynamics, so that antepartum visits are now designated as high risk with resident involvement and midwives have become part of a larger team.

Most of the graduated residents have made the decision to remain in academic medicine and help train and mentor the incoming residents. This portends well for improving health workforce capacity and sustainability. One of the residents who was admitted into the program in 2016, entered an IGCS supported 2-year fellowship program in gynecologic oncology at the Uganda Cancer Institute in Kampala after graduating in 2019, and is scheduled to return to Liberia in 2023.

### Lessons Learned

*Establishing a local training program provided the opportunity to train more specialists than the country would have done if candidates were sent outside the country*

*Training locally also prevented depletion of the few physicians left in the country providing health care*.

*Establishment of the Liberian College of Physicians and Surgeons strengthened Liberian physicians' participation in the West African Colleges of Physicians and Surgeons*

*Annual budgeting for faculty posed additional challenges at the end of an academic year and the beginning of the next one, causing delays in starting the academic year and uncertainty for retaining the same faculty members*.

*Strong partnerships were built between the participating institutions*.

September 2017 marked the inaugural graduation of Liberian trained specialists. The Obstetrics and Gynecology residency faced major obstacles, but the program prevailed with the help of multi-institutional support. The graduation marked the beginning of a pipeline of specialists dedicated to improving maternal care in the country. In 2017, the World Bank, through the Ministry of Health funded a program with US Academic Partners that allowed hiring of sub-regional faculty to support training of residents in all four specialties. The Liberian training program today has expanded to include additional residency training in Family Practice, Psychiatry and Ophthalmology. All seven (7) residency programs have continued without interruption during the COVID-19 pandemic. Six cohorts of Ob-Gyn residents have since graduated increasing the number of Obstetrics and Gynecology specialists in the country.

## Conceptual or Methodological Constraints

Initially, the programs were established as a 4-year residency that was based not at one hospital, but several hospitals throughout the country. This was to ensure that rural communities had access to specialists as part of a primary care team. Over the course of the first year however, it became clear that the infrastructure in most of the rural hospitals was not adequate to support training, and the program became centered at JFK Hospital in Monrovia. The duration of the program was also decreased from 4-years to 3 years because of the delay in the first year of training due to Ebola and the urgent need for specialists in the country.

The Ebola virus outbreak was a catastrophic challenge to the program. The first confirmed case of Ebola was in Lofa County near the border with Guinea in March 2014. Sporadic cases occurred in rural areas through early May 2014 with little impact on the residency program. By late May however, a second wave of Ebola included the first documented case in Monrovia in a patient who had traveled through the city, changing the outbreak from a rural to an urban epidemic (21). During that period residency training was suspended for 9 months and residents were deployed as front-line workers to assist in the response. During the deployment one resident contracted Ebola and succumbed to his infection, one of 184+ health workers who died during the outbreak. When residents resumed training in June 2015, the schedule was adjusted to shift the training to Bong County where there were only sporadic cases of Ebola. Liberia was not declared Ebola free until June 2016.

It was difficult to provide objective data demonstrating real time improvements due to the residency program. Liberia like most African countries has challenges in data collection, accuracy, and consistency. Based on data retrieved from “Morning Report,” the number of referrals, and the volume of patients attending JFK antepartum clinic, have shown a steady increase over the course of 3-years, indicating increasing trust from the surrounding communities. Another measure that has been demonstrated through Morning Report, reflects an almost 40% decrease in primary Cesarean section. Because JFK Hospital is a referral hospital many of the Cesarean sections performed were on patients who were sent as emergencies after hours or days of labor at home or in community settings. This accounted for an estimated 50–70% Cesarean section (C-section) rate. The noticeable decrease in C-section rates, reflects better communication with the community and earlier admission to the hospital as well as improved judgement and recognition of true emergencies by the resident staff. As the program continues to grow and the administrative assistance to support the residency programs increases, a focus should be on improving data collection as a means of improving decision making and impacting patient outcomes.

## Data Availability Statement

The original contributions presented in the study are included in the article/supplementary material, further inquiries can be directed to the corresponding author/s.

## Author Contributions

AMB, MR, AB, LR, JM, JJ, VK, MB, SS, BJ, RM, and BD contributed to conception and design of the study. ML, AD, CM, SC, JS, EG, TN, and WL contributed to carrying out the study. All authors have contributed by drafting sections of the manuscript, editing, and approving the final version that is currently being submitted.

## Conflict of Interest

The authors declare that the research was conducted in the absence of any commercial or financial relationships that could be construed as a potential conflict of interest.

## Publisher's Note

All claims expressed in this article are solely those of the authors and do not necessarily represent those of their affiliated organizations, or those of the publisher, the editors and the reviewers. Any product that may be evaluated in this article, or claim that may be made by its manufacturer, is not guaranteed or endorsed by the publisher.
